# Targeting FBPase is an emerging novel approach for cancer therapy

**DOI:** 10.1186/s12935-018-0533-z

**Published:** 2018-03-09

**Authors:** Gao-Min Liu, Yao-Ming Zhang

**Affiliations:** Department of Hepatobiliary Surgery, Meizhou People’s Hospital, No. 38 Huangtang Road, Meizhou, 514000 China

**Keywords:** FBPase, Cancer, Clinical significance, Mechanism

## Abstract

Cancer is a leading cause of death in both developed and developing countries. Metabolic reprogramming is an emerging hallmark of cancer. Glucose homeostasis is reciprocally controlled by the catabolic glycolysis and anabolic gluconeogenesis pathways. Previous studies have mainly focused on catabolic glycolysis, but recently, FBPase, a rate-limiting enzyme in gluconeogenesis, was found to play critical roles in tumour initiation and progression in several cancer types. Here, we review recent ideas and discoveries that illustrate the clinical significance of FBPase expression in various cancers, the mechanism through which FBPase influences cancer, and the mechanism of FBPase silencing. Furthermore, we summarize some of the drugs targeting FBPase and discuss their potential use in clinical applications and the problems that remain unsolved.

## Background

Cancer is one of the leading causes of death worldwide [1]. In the past decade, reprogramming of energy metabolism has emerged as new hallmarks of cancer [2]. There is increasing epidemiological evidence that links the risk of cancer with metabolic disorders, such as diabetes and obesity [3]. However, the complete regulatory network of metabolism reprogramming remains to be elucidated.

Glucose homeostasis is reciprocally controlled by the catabolic glycolysis and anabolic gluconeogenesis pathways. Under normoxic conditions, normal cells process glucose first to pyruvate via glycolysis in the cytosol and thereafter to carbon dioxide in the mitochondria (oxidative phosphorylation, OXPHOS); under anaerobic conditions, glycolysis is favoured, and relatively little pyruvate is dispatched to the oxygen-consuming mitochondria [4, 5]. However, as first observed by Otto Warburg in the 1920s, some cancer cells preferentially rely on glycolysis, even in conditions of high oxygen tension (‘‘aerobic glycolysis” or “the Warburg effect’’) [6, 7]. Aerobic glycolysis was validated with the wide use of ^18^F-deoxyglucose positron emission tomography (FDG-PET) in most cancers [8, 9]. Aerobic glycolysis leads to several advantages for tumour growth by increasing glucose intake, enhancing lactate production and secretion, and diverting glycolytic intermediates to anabolic reactions such as ribose synthesis, serine and glycine synthesis, phosphoglycerol synthesis, and protein glycosylation [10]. Moreover, recent studies have developed promising individualized therapeutic strategies by targeting the altered energy metabolism for the treatment of cancer [11].

Though our understanding of metabolic reprogramming in cancer is progressing at an unprecedented pace, previous studies have mainly focused on glycolysis. Recently, fructose-1,6-bisphosphatase (FBPase), a rate-limiting enzyme responsible for gluconeogenesis, was found to play critical roles in tumour initiation and progression in several cancer types [12–28]. Here, we search Pubmed, EMbase, Web of Science using keywords “FBPase” and “cancer” (the most recent published report search date: 1 August 2017) and review recent ideas and discoveries regarding the role of FBPase in cancer.

## FBPase location and regulation

**Fig. 1:**
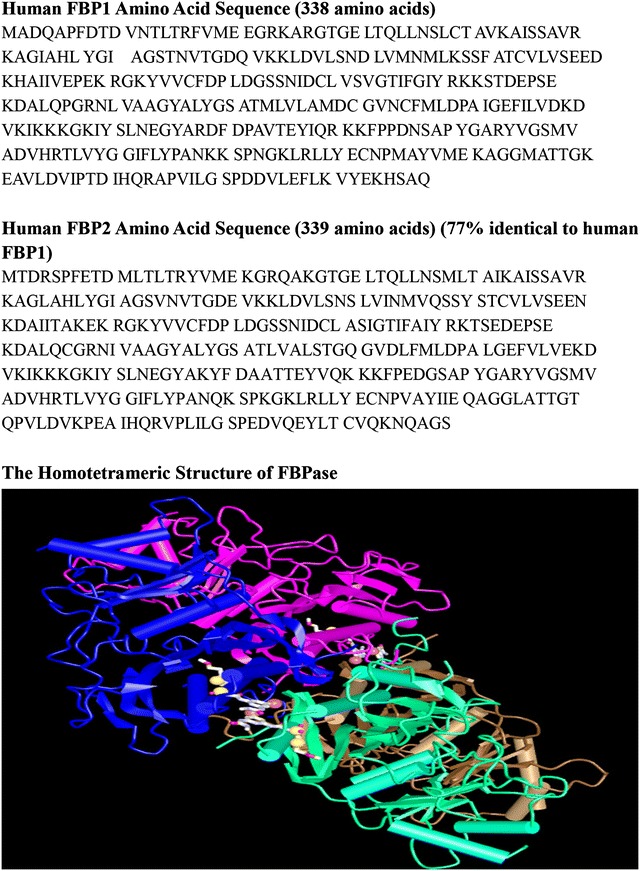
Amino acid sequences and characteristic homotetrameric structure of human FBPase. This figure provides the human FBP1 and FBP2 amino acid sequence. Human FBP2 is 77% identical to human FBP2 [34]. The sequence alignments were run through BLAST. The characteristic homotetrameric structure of human FBPase was shown [35]

FBPase (EC 3.1.3.11), which catalyses the hydrolysis of fructose 1,6-bisphosphate to fructose 6-phosphate and inorganic phosphate, is a rate-limiting enzyme responsible for gluconeogenesis and glyconeogenesis and, more generally, for the control of energy metabolism and glucose homeostasis [29]. In mammals, two separate genes, FBP1 and FBP2, encode the liver and muscle isoforms of FBPase, respectively. The FBP1 gene, which is mainly expressed in gluconeogenic organs, such as the liver and kidneys, is located at chromosome region 9q22.3. FBP1 consists of seven exons which span more than 31 kb that encode 338 amino acids and six introns [30]. The FBP1 promoter has been well characterized since 2000 [31]. The FBP2 gene, which was initially isolated from muscle tissues and later found to be expressed in all cells, is located at chromosome region 1p36.1 [32]. FBP2 encodes a 339-amino acid protein that shares 77% identity with the FBP1 protein [33]. However, the promoter of the FBP2 gene has not yet been characterized. All FBPases are homotetrameric proteins with a molecular weight of approximately 37 kDa per subunit (Fig. 1). FBP1 has been identified as the regulatory enzyme of gluconeogenesis, but the physiological role of FBP2 is far from clear.

Acute regulation of FBPase is achieved mainly through its oscillation between two conformational states: the inactive T form when in complex with AMP and the active R form [29, 36–40]. By modulating pyruvate kinase activity, fructose-1,6-bisphosphate (F1,6BP) affects substrate cycling in the pyruvatel/phosphoenolpyruvate (PEP) substrate cycle [41]. Phosphofructokinase-1 (PFK1), the “gatekeeper” of glycolysis, converts fructose-6-phosphate (F6P) to F1,6BP and has the opposite effect of FBPase in regulating F1,6BP [42]. Fructose-2,6-bisphosphate (F2,6BP) is a potent allosteric activator of PFK1 and a competitive inhibitor of FBPase [5, 41–44]. When the level of F2,6BP is low, the rates of gluconeogenesis are high (such as in starvation and diabetes). Conversely, when the level of F2,6BP is high, the rate of gluconeogenesis is low (such as during refeeding and insulin administration) [5]. The synthesis and hydrolysis of this regulator are catalysed by the bifunctional enzyme 6-phosphofructo-2-kinase/fructose-2,6-bisphosphatase (PFK-2/FBPase-2) [45]. Phosphorylation of this enzyme by cAMP-dependent protein kinase results in inhibition of the kinase and activation of the bisphosphatase, whereas dephosphorylation has the opposite effect [36, 40]. TIGAR (TP53-induced glycolysis and apoptosis regulator), a recently identified enzyme, shares similarities with the bisphosphatase domain of PFK-2/FBPase-2 [46]. TIGAR acts to degrade intracellular F2,6BP [47]. Interestingly, FBP1 and FBP2 differ significantly in their kinetic properties. FBP2 is approximately 100 times more susceptible to the allosteric inhibitors AMP and NAD+ [48] and approximately 1000 times more sensitive to inhibition by Ca^2+^ than FBP1 [49, 50]. Crystal structures have shown that in contrast to the well-studied R form of FBP1, which is flat, the R form of FBP2 was diametrically different, with a perpendicular orientation of the upper and lower dimers [37].

**Fig. 2:**
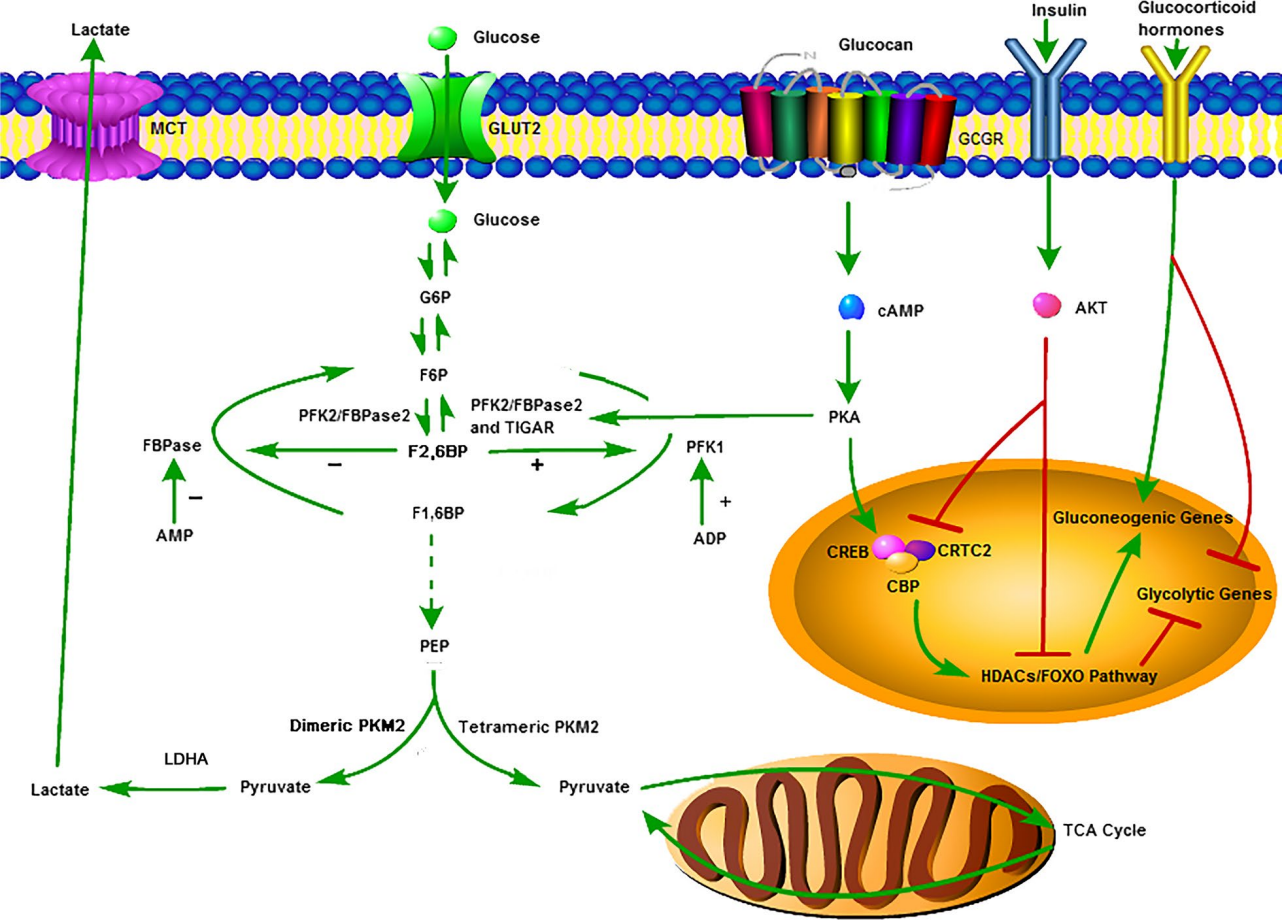
Regulation of FBPase. Acute regulation of FBPase is achieved mainly through allosteric regulator. Chronic regulation of FBPase occurs through transcriptional and hormonal regulation mainly by glucagon, glucocorticoid hormones and insulin. *ADP* adenosine diphosphate, *AMP* adenosine monophosphate, *AKT* protein kinase B, *cAMP* cyclic adenosine monophosphate, *CBP* CREB-binding protein, *CREB* cAMP response element-binding protein, *CRTC2* CREB coactivator, *G6P* glucose-6-phosphate, *GLUT2* glucose transportor 2, *GCGR* glucagon receptor, *F1,6BP* fructose-1,6-bisphosphate, *F2,6BP* fructose-2,6-bisphosphate, *F6P* fructose-6-phosphate, *FBPase* fructose-1,6-bisphosphatase, *FOXO* forkhead box O protein, *HDAC* histone deacetylase, *LDHA* lactate dehydrogenase A, *MCT* monocarboxylate transporters, *PFK2/FBPase2* 6-phosphofructo-2-kinase/fructose-2,6-bisphosphatase, *PEP* phosphoenolpyruvate, *PFK1* phosphofructokinase-1, *PKA* protein kinase A, *PKM2* pyruvate kinase M2, *TIGAR* TP53-induced glycolysis and apoptosis regulator, *TCA* tricarboxylic acid

Chronic regulation of hepatic glucose metabolism occurs through transcriptional and hormonal mechanisms [51]. The master hormone that promotes gluconeogenesis is glucagon. Increased glucagon stimulates gluconeogenesis via the induction of intracellular cAMP. cAMP triggers protein kinase A (PKA), leading to the phosphorylation of PFK-2/FBPase-2, which activates FBPase-2, leading to the dephosphorylation of F2,6BP and concomitant increases in FBPase activity and gluconeogenesis [45]. In addition, PKA activates cAMP response element-binding protein (CREB), the CREB coactivator CRTC2 [52], and the class II histone deacetylases (HDACs)/FOXO pathways [53, 54] and promotes the expression of key gluconeogenic genes; however, it suppresses the expression of glycolytic genes. Glucocorticoid hormones and glucagon plays essential synergistic roles in the regulation of gluconeogenesis [55]. The response to glucocorticoids is mediated by the glucocorticoid receptor (GR), which binds to glucocorticoid responsive elements (GREs) in the promoters of gluconeogenic genes [56]. In contrast, insulin acts to repress the transcription of gluconeogenic enzymes. This repression of the expression of gluconeogenic enzymes is achieved via the activation of the insulin-phosphoinositide 3-kinase (PI3K)-Akt pathway or suppression of the cAMP/PKA/CREB pathway [57] (Fig. 2).

## FBPase and non-cancerous diseases

### FBP1 deficiency

Mutations in the FBP1 gene cause FBP1 deficiency, an inherited autosomal recessive disorder, which leads to the impairment of glucose synthesis from all gluconeogenic precursors [58]. This deficiency was first described by Baker and Winegrad in 1970 [59]. This disorder is characterized by recurrent episodes of hypoglycaemia and metabolic acidosis during fasting, with symptoms usually manifesting during the first days of life [60–62]. If not treated appropriately, FBP1 deficiency leads to unexpected infant death [63]. However, with diet control and avoidance of prolonged fasting, most adult patients exhibit relatively normal clinical profiles.

### FBPase and type 2 diabetes

Blood glucose levels are elevated in type 2 diabetes (T2DM) due to impaired insulin secretion resulting from declining β-cell function; decreased glucose uptake by tissues such as muscle, liver, and fat; and increased hepatic glucose production (HGP) [64]. Gluconeogenesis contributes approximately 50% of the total HGP in humans following overnight fasting and is primarily responsible for the increase in fasting HGP in individuals with T2DM [64–66]. The rate-limiting enzymes of gluconeogenesis have been raised as potential targets for combating T2DM. FBPase is an attractive target as it functions within only the gluconeogenesis pathway [67]. In animal models, the inhibition of FBPase markedly inhibited gluconeogenesis and increased glucose sensitivity and utilization [68]. Upregulation of FBPase in pancreatic islet cells, as examined in transgenic mice or stably transfected pancreatic cell lines and occurring in states of T2DM, decreased the cell proliferation rate and significantly suppressed glucose-induced insulin secretion (GSIS) [69]. Downregulation of FBP1 in mouse pancreatic β-cells by small interfering RNA enhanced glucose utilization and GSIS, whereas overexpression of FBP1 decreased GSIS [70]. Phase 2 clinical studies of some inhibitors of FBP1in T2DM are in progress [71–73].

## FBPase and cancer

Accumulating evidence has disclosed the role of FBPase in the carcinogenesis, development and progression of various cancer types. Lower FBPase expression frequently correlated significantly with an advanced tumour stage, a highly malignant phenotype, and worse prognoses in cancer patients. All these data implied that FBPase might be a novel biomarker and potential target for the treatment of cancer (Table 1).

**Table 1:** FBPase expression in cancers (listed in alphabetical order)

Type of cancer	FBPase expression	Change in expression over disease progression	Prognostic significance	Reference(s)
Breast cancer	Lower in animal model, human breast cancer [74–76], basal-like breast cancer cell lines [20], triple-negative breast cancer but not in luminal cell lines [19] and brain metastatic cells [21]. Data mining shown FBP1 over-expression were common in breast cancer irrespective of histological type in cell lines and human breast cancer [20]	Expression inhibited tumorigenicity in vitro and tumor-formation in vivo [20, 22] but promoted the growth of brain metastasis [21]. FBP1 expression associated with nuclear grade and tumor stage [18]	Loss of FBP1 expression associated with poor survival [18, 20, 22]. But data mining shown no correlation between FBP1 and prognosis in triple-negative breast cancer [20]	[18–22, 74–76]
Colon cancer	Lower in cancer cell lines and in human colon cancer [17]	Overexpression reduced cancer cell colony formation and inhibited the growth of cancer cells [17]		[17]
Gastric cancer	Downregulated in gastric cancer cell lines and gastric carcinomas [17, 25, 26]	Overexpression inhibited proliferation inhibition in vitro as well as xenograft tumor growth in vivo [25, 26]	Absent or low FBP2 expression correlated with poor survival [25]	[17, 25, 26]
Liver cancer	Decreased in 3-methyl-4-dimethyl aminoazobenzene (3MeDAB) induced [77] and choline-deficient diet-induced hepatocellular carcinoma model [78]; Decreased in most human liver cancer cell lines [14, 17] and in human hepatocellular carcinoma [15–17, 77, 79–82]	Low expression correlated with highly malignant phenotype, including large tumor size, poor differentiation, advanced tumor stage [15, 80–82], vascular cell invasion and high pathological grade [14]	Loss of FBP1 expression associated with poor overall survival and higher tumor recurrence rates [14, 15, 79, 81, 82]	[14–17, 77–82]
Lung cancer	Loss in lung cancer cells [12, 13] and in human lung cancer tissues [13, 83, 84]	Forced expression inhibited tumorigenesis and invasion in lung cancer cells [12, 13] and cancer progression in human lung cancer [13]	Low FBP1 expression correlated with poor overall survival [13]	[12, 13, 83, 84]
Pancreatic cancer	Lower in pancreatic cancer tissues [27, 28]		FBP1 expression inversely correlated with tumor grades and prognosis [27, 28]	[27, 28]
Renal carcinoma	Ubiquitous loss in clear cell renal cell carcinoma [23, 24, 85, 86]	FBP1 expression in several renal cancer cell lines inhibited their growth. Suppression of FBP1 correlated with advanced tumour stage [24]. But no correlation was found between clinicopathological factors, including age, gender, T stage, Fuhrman grade and expression of FBP1 expression in another study [23]	Suppression correlated with worse patient prognosis [24]	[23, 24, 85, 86]
Small intestinal neuroendocrine tumour	Comprehensive integrated genomic analysis shown epigenetically dysregulation [87]			[87]

### Breast cancer

FBPase was found to decrease significantly in animal models, basal-like breast cancer (BLBC) cell lines, triple-negative breast cancer (TNBC), and human breast cancer but not in luminal cell lines and brain metastatic cells [19, 21, 22, 74–76]. FBPase inhibits tumourigenicity in vitro and tumour formation in vivo [20, 23] as well as the growth of brain metastasis [21]. FBP1 expression was associated with the nuclear grade and tumour stage [18]. Loss of FBP1 expression was associated with poor survival [18, 20, 22] and was strongly related to palindromia after tamoxifen treatment in patients with breast cancer [88].

However, some contradictory results existed. FBP1 over-expression was found to be a common event, irrespective of histological type, in cell lines and human breast cancer. Furthermore, there was no correlation between FBP1 and the prognosis of TNBC [20]. These contradictory findings indicated that the prognostic value of FBP1 in breast cancer may be molecular type- and tissue type-dependent.

### Gastric cancer

Expression of FBP1 and FBP2 was significantly downregulated in gastric cancer cell lines and gastric carcinomas (GCs) due to promoter hypermethylation [17, 25, 26]. Ectopic expression of FBPase in GC cells led to significant inhibition of proliferation in vitro, as well as xenograft tumour growth in vivo [25, 26]. Absent or low FBP2 expression [25] and FBP1 promoter methylation [26] in GC tissues was correlated with the poor survival of GC patients. However, no significant correlation between the methylation of the FBP1 promoter and clinicopathological features such as age, gender, Helicobacter pylori status, Lauren type, differentiation or pathologic stage was found [26].

### Liver cancer

FBPase suppression in liver cancer has been widely validated in animal models [77, 78], cancer cell lines [14, 17] and clinical specimens [14, 15, 17, 24, 78–82]. Restoration of FBP1 expression by agents such as plumbagin [77], 5-aza-2′-deoxycytidine (Aza) [17], dexamethasone [89], or bortezomib [80] or by ectopic lentiviral transfection [14] could significantly inhibit cell growth and colony-formation ability in vitro [14, 17, 80], as well as xenograft tumour growth in vivo [15, 81, 89]. Hepatocellular carcinoma (HCC) exhibiting low expression of FBP1 had a highly malignant phenotype, including large tumour size, poor differentiation, and advanced tumour stage [15, 80–82], as well as vascular cell invasion and a high pathological grade (stages III–IV) [14]. Loss of FBP1 expression was associated with poor overall survival and higher tumour recurrence rates [14, 15, 79, 81, 82].

In addition, proteomic techniques have recently provided new evidence. Proteomic analysis has revealed significant downregulation of FBPase in carcinogenic processes in rats after as early as 3 weeks of exposure, indicating its potential utility as an early predictive biomarker for liver carcinogenicity [90]. In a review including a total of 16 proteomic studies, FBP1 was one of 27 proteins identified as differentially expressed proteins with consistent directions of change in at least three studies, that were found to be potential biomarkers for HCC [91].

### Lung cancer

FBP1 was absent in lung cancer cells, and forced expression of FBP1 led to inhibition of tumorigenesis and invasion, especially under hypoxic conditions (0.1% oxygen) [12, 13]. In human lung cancer tissues, FBP1 mRNA and proteins were found to poorly express when compared to paired normal lung tissues [13, 83, 84]. Low FBP1 expression correlates with poor overall survival and cancer progression [13].

### Renal carcinoma

Recently, a ubiquitous loss of FBP1 expression has been identified in clear cell renal cell carcinoma (ccRCC) [23, 24, 85, 86]. Ectopic FBP1 expression in several ccRCC cell lines significantly inhibited their growth, while FBP1 depletion promoted the growth of kidney proximal tubule cells, the presumptive cells-of-origin for ccRCC. Lower FBP1 expression correlated significantly with advanced tumour stage and worse patient prognosis [24]. However, in another study, no correlation was found between clinicopathological factors, including age, gender, T stage, and Fuhrman grade and the expression of FBP1. This finding may be partially due to the high proportion of patients with low T stages and low Fuhrman grades in that cohort [23].

### Other cancer types

There is an even bigger knowledge gap in the role of FBPase in some cancer types due to only one to two publications, such as in colon cancer, pancreatic cancer and small intestinal neuroendocrine tumor.

Downregulation of FBP1 was also observed in HT29, SW480, SW620, HCT116, LoVo and RKO colon cancer cell lines when compared to human normal adult colon tissue, and this downregulation correlated well with the promoter methylation status of FBP1. FBP1 overexpression reduced the colony formation abilities of cancer cells and inhibited their growth. In human colon cancer, FBP1 expression was significantly downregulated in 80% (4/5) of the colon tumour tissues when compared with adjacent non-tumour tissues [17].

Recently, one study reported their data of integrated molecular analysis of small intestinal neuroendocrine tumour (SINETs) in 97 tumours and 25 normal tissue samples from 85 individuals. 21 epigenetically dysregulated genes were identified, one of the most significant genes was FBP1 (84%). 82% of tumour specimens demonstrate altered methylation in at least 4 of the top 5 frequent altered candidates including caudal type homeobox 1 (CDX1), FBP1, transmembrane protein 171 (TMEM171), ganglioside induced differentiation associated protein 1 like 1 (GDAP1L1), and cadherin, EGF LAG seven-pass G-type receptor (CELSR3) [87].

FBP1 was consistently reported to be expressed at low levels in pancreatic cancer tissues; on the contrary, FBP1 was always at high level in normal pancreatic tissues. FBP1 expression inversely correlated with tumour grade and prognosis [27, 28].

## Mechanism through which FBPase influences cancer

**Fig. 3:**
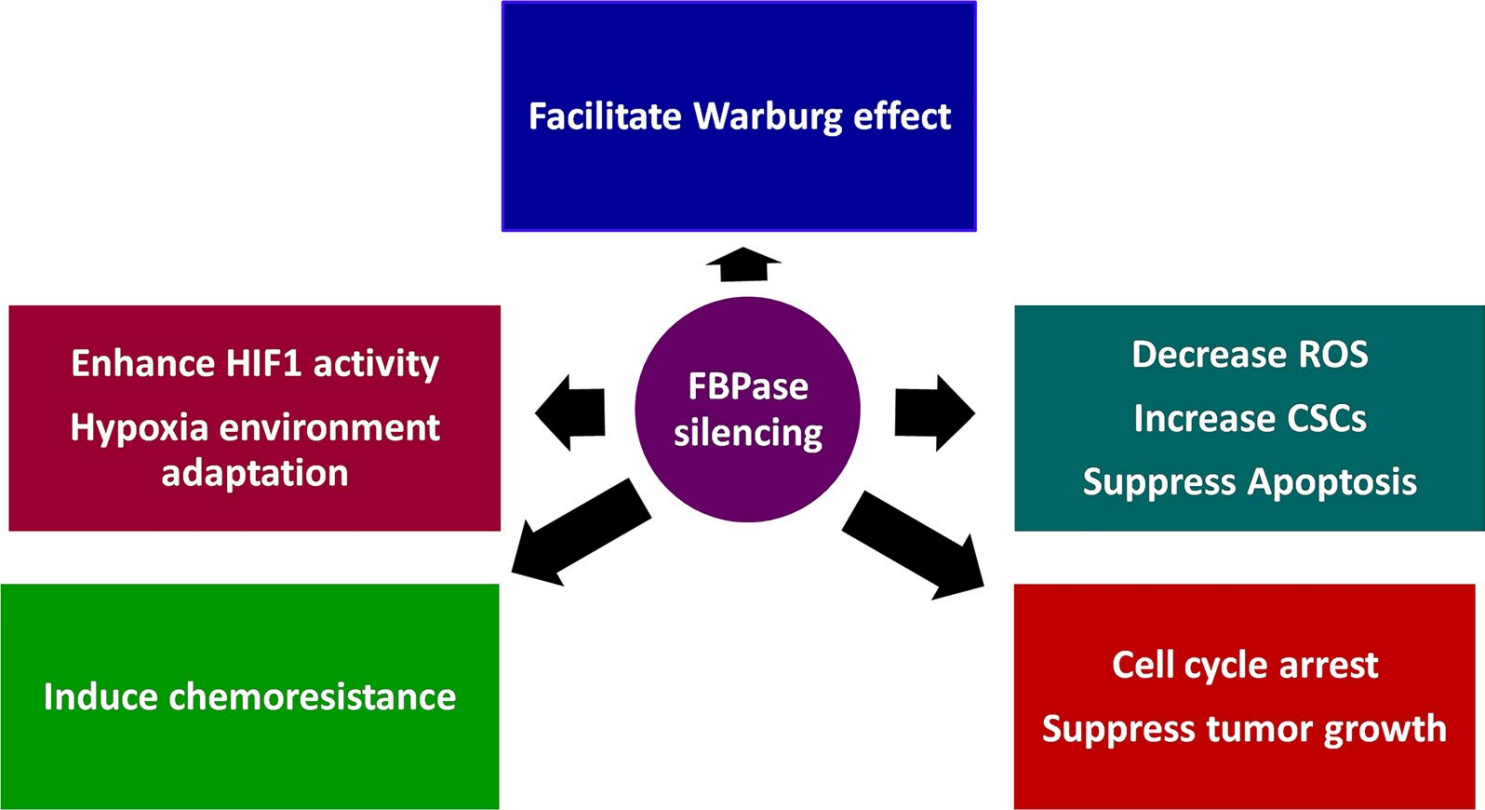
Mechanisms through which FBPase influence cancer

The mechanism through which FBP1 influences cancer cells were summarized in Fig. 3. At present, the role of FBP1 in regulating the Warburg effect in cancer cells was best understood.

### Loss of FBPase facilitates the Warburg effect in cancer

Glucose uptake and lactate secretion are two common indicators of glycolysis. Upon FBPase silencing, glucose uptake and lactate secretion were significantly increased in various cancer cells (BLBC [20, 22], ccRCC [24], gastric cancer [26], HCC [14, 15, 80–82], lung cancer [12, 13], and pancreatic cancer cell lines [28]). TXNIP, a commonly used intracellular glucose sensor [92], and insulin, the major hormone regulating glucose uptake [53], were tested in cancer cell lines. FBP1 expression decreased glucose uptake, TXNIP induction and insulin sensitivities, whereas the loss of FBP1 enhanced glucose uptake, TXNIP induction and insulin sensitivities [13, 16, 23, 24]. These finding indicated that FBP1 was critical in inhibiting glucose uptake, as exemplified by the downregulation of glucose and insulin sensitivities. Accumulation of lactate is another common feature of cancer cells and is involved in the progression of malignancies [93]. Lactate secretion was significantly decreased in FBP1-expressing cells [12, 13, 15, 22, 26, 80]. The extracellular acidification rate (ECAR) is the glycolysis rate after glucose treatments and is equal to the glycolysis capacity after oligomycin treatment. In HCC, studies have shown that FBP1 significantly reduced the ECAR, while FBP1 suppression did the opposite [14].

Besides, certain regulators in carcinogenesis were found to reprogram cancer cell metabolism by suppressing FBP1. NMP1, a multifaceted nucleolar protein, was found to stimulate glucose uptake and lactate generation in pancreatic cancer cells by directly inhibiting FBP1 expression. Restoring FBP1 in pancreatic cancer cells reversed the NPM1-induced dysfunction of glucose metabolism [28]. TRIM proteins, members of a subfamily of the RING type E3 ubiquitin ligases [94], were found to increase glucose consumption and lactate production in HCC cells by promoting FBP1 degradation. Importantly, the effect of TRIM28 was largely inhibited by the co-expression of FBP1 [80]. c-Myc, a crucial downstream factor of Wnt/β-catenin signalling, was found to be negatively correlated with the level of FBP1 in breast cancer cells [20]. As c-Myc is also a transcription factor involved in metabolic reprogramming [95], it is reasoned that the inhibitory effect of FBP1 in glycolysis might be mediated partially by the downregulation of c-Myc. However, the precise mechanism by which FBP1 regulates Wnt/β-catenin signalling warrants further investigation [20].

In addition, the consuming oxygen during OXPHOS and aerobic glycolysis is totally different. The reliance of tumour cells on glycolysis for energy production causes them to decrease oxygen consumption to adapt to the hypoxia tumour microenvironment. The basal oxygen consumption rate (OCR) was found to be significantly decreased in FBP1-knockdown cells, whereas it was significantly increased in FBP1-expressing cells. Similar results were obtained in the analyses of ATP-linked and maximal OCR [12, 13, 22], indicating that FBP1 is involved in the switch from glycolysis to OXPHOS.

FBPase silencing helped maintain energy homeostasis in cancer cells. For every glucose molecule a cell consumes, aerobic glycolysis produces 2 ATP, whereas OXPHOS produces 36 ATP. Under normoxic conditions, expression or knockdown of FBP1 did not alter the steady-state level of ATP in BLBC or luminal cells. However, under hypoxia, knockdown of FBP1 helped maintain ATP production, whereas expression of FBP1 significantly decreased ATP production in BCLC and HCC [15, 22]. In gastric cancer cells, studies have found that FBP2 overexpression significantly reduces the levels of ATP and lactate through interference of the Akt-mTOR pathway [25].

Stable isotope-resolved metabolomic (SIRM) analysis was used to investigate the metabolic fate of [U-^13^C]-glucose, which directly produces glycolytic intermediates that contain six or three ^13^C atoms (M6/M3 species) and the intermediates of the first turn of the tricarboxylic acid (TCA) cycle that contain two ^13^C atoms (M2 species). When FBP1 was overexpressed, M3 enrichment of lactate was significantly inhibited, and the levels of the glycolytic intermediates F-1,6-BP (M6 species), dihydroxyacetone phosphate (M3 species), and glucose-6-phosphate were decreased. Ectopic FBP1 tended to inhibit M2 enrichment of TCA cycle intermediates, such as succinate, fumarate and malate, as well as M4 enrichment of citrate [15, 22, 96]. Furthermore, FBP1 expression reduced M5 enrichment of the ribosyl unit of ribonucleotides and their derivatives (i.e., NAD+, and UDPG), suggesting that FBP1 suppressed de novo nucleic acid synthesis through the pentose phosphate pathway [15, 22, 24]. In line with these findings, the ratio of NADP+/NADPH was increased in FBP1-expressing cells, whereas this ratio was decreased in FBP1-knockdown cells. The production of the M3 isotopologues of glycerol-3-phosphate (G3P) and serine was significantly reduced in FBP1-expressing cells [22]. Reduced glucose-dependent TCA flux is known to increase anaplerotic glutamine flux [97]; elevated glutamine uptake and enrichment of glutamine-derived TCA cycle intermediates (M4 species) were observed upon forced FBPase expression [21, 24]. Increased oxidation of branched chain amino acids (BCAAs) (valine, leucine, and isoleucine), except for glutamine, was also found in brain metastatic cancer cells, with upregulation of FBP2, but not FBP1, and enhanced gluconeogenesis in the absence of glucose [21].

All these data imply that loss of FBPase facilitates glycolytic flux and decreases OXPHOS in cancer cells.

### How cancer cells switch OXPHOS to aerobic glycolysis by FBPase silencing

Key enzymes of glucose uptake and aerobic glycolysis in cancer cells were found to decrease significantly when FBPase was expressed. In HCC, FBP1 expression was found to significantly decrease the levels of glucose transporter 1 (GLUT1) and lactate dehydrogenase A (LDHA) [82]. Three enzymes are involved in catalysing the irreversible steps of glycolysis: hexokinase (HK) [98, 99], phosphofructokinase (PFK) [100], and pyruvate kinase (PK) [101]. While dimeric PKM2 diverts glucose metabolism towards anabolism through glycolysis, tetrameric PKM2 promotes the flux of glucose-derived carbons for ATP production via oxidative phosphorylation [101]. FBP1 expression significantly decreased the tetrameric PKM2, whereas knockdown of FBP1 increased the formation of tetrameric PKM2. In addition, FBP1 expression was found to significantly decrease the HK2 and PFK1 levels in HCC [15]. All these results indicated that the loss of FBP1 activated GLUT1, PKM2, HK2, PFK1 and LDHA, which facilitated glucose uptake and lactate production and triggered the switch to aerobic glycolysis.

In addition, FBP1 expression was found to be correlated with higher complex I activity. TFB1M is a nuclear gene, encoding mitochondrial transcription factor, which is essential for mitochondrial biogenesis and OXPHOS [102]. When FBP1 was expressed, TFB1M and its targets from mitochondrial complex I, ND1 and ND5, were found to be increased, indicating that the increase in complex I activity is the main factor underlying the increase in OXPHOS [22].

## FBPase increased ROS generation, reduced cancer stem cells, and induced apoptosis

Along with increases in complex I activity and mitochondrial OCR, ROS levels increased when FBPase was expressed [12, 17, 19, 22, 25]. ROS amplify tumourigenic phenotypes, such as cancer stem cells (CSCs) [103]. Breast CSCs are enriched in cells with a CD44 high/CD24 low/EpCAM+ phenotype [104]. In BLBC cell lines, FBP1 expression significantly reduced the percentage of CD44 high/CD24 low/EpCAM+ populations and decreased tumour sphere formation. Conversely, FBP1 silencing resulted in an increased CSC-like phenotype [22]. Mechanically, increased ROS levels induced by FBP1 would shift the interaction of β-catenin from TCF4 to FOX O3a and thus inhibit tumourigenicity in vitro and tumour formation in vivo.

Moreover, ROS induces mitochondrial apoptosis [105]. Forced FBP1 expression in lung cancer stem cells and breast cancer or FBP2 expression in gastric cancer increased apoptosis by reducing the Bax/Bcl-2 ratio, inducing poly ADP-ribose polymerase (PARP), caspase-3 and caspase-9 activation and suppressing endogenous ROS scavenging systems such as superoxide dismutase (SOD) [12, 19, 25]. In breast cancer cells, FBP1 limited the efficient removal of diseased mitochondria and reduced the expression of hypoxia-induced factor 1α (HIF1α), BCL2/adenovirus E1B 19 kDa interacting protein 3-like (BNIP3L/NIX), and BCL2/adenovirus E1B 19 kDa interacting protein 3 (BNIP3), which disrupts BNIP3/NIX-Bcl-2 complex formation under normal conditions but promotes complex formation between Bcl-2 and Beclin 1 [19]. Annexin V+/propidium iodide (PI)− and Annexin V+/PI+ cells represent early apoptotic cells and late apoptotic/necrotic cells, respectively [106]. FBP2 expression increased both subpopulations in gastric cancer cells [25]. In brain metastatic breast cancer cells, knocking down FBP2 resulted in a significant amount of apoptotic cell death (as indicated by the increase incleaved PARP), whereas exogenous FBP2 significantly rescued cell death [21].

## FBPase induced cell cycle arrest to suppress tumour growth

The ability to sustain chronic proliferation is the most fundamental trait of cancer cells. Normal tissues carefully maintain the homeostasis of cell number, normal tissue architecture and function by rigorous regulation of progression signals through the cell cycle as well as cell growth. But cancer cells, by hijacking these regulating mechanisms, become masters of their own destinies [2]. Cell cycle arrests if the numbers of cells in G2 and M phase increase, whereas it proceeds if the numbers of cells in S phase increase. Cell cycle distribution was investigated to understand the molecular mechanisms by which FBP1 suppressed colony formation and cell proliferation. In hepatocellular carcinoma and colon cancer, forced FBP1 expression was found to increase the number of G2–M phase cells but decrease the number of S phase cells [17]. In lung cancer, FBP2 accumulated in cell nuclei during the S and G2 phases and interacted with histone family members and with several proteins involved in cell-cycle regulation and RNA processing [83, 107]. All these data indicated that the growth suppression induced by forced FBP1 expression might be partly due to cell cycle arrest. However, the detailed mechanism remains unclear.

## FBPase inhibits HIF1 activity and influences adaption to hypoxia tumour microenvironment of cancer

All cancer cells suffer hypoxia microenvironment due to rapid proliferation, differentiation and poorly formed tumour vasculature [108]. HIF1, a well-known transcriptional regulator, is the most important aspect of how cancer cells respond to the unfriendly microenvironment. HIF1 promotes glycolytic metabolic alterations by the activation of multiple glycolytic genes. Under conditions of persistent hypoxia, the induction of HIF1 leads to adaptive mechanisms for the reduction of ROS and re-establishment of homeostasis [109].

Knockdown of FBP1 significantly reduced growth inhibition in luminal cell lines under hypoxic condition (0.1% O_2_) but not at normoxic condition (21% O_2_). Similarly, FBP1 expression induced a drastic growth inhibition in BLBC cell lines under hypoxic condition but not at normoxic condition [22]. The expression of FBP1, but not of FBP2, was inversely correlated with HIF1α activity in RCC [23, 24]. FBP1 was also found to be negative correlated with HIF1α activity in HCC and breast cancer [18, 82]. Ectopic expression of FBP1 suppressed HIF activity and reduced the expression of HIF target genes, whereas loss of FBP1 enhanced HIF activity [24]. FBP1 inhibited HIF1α activity in the nucleus via a direct interaction with the HIF-inhibitory domain in an enzyme-activity-independent manner [24]. ALL these results indicated that the regulation of FBP1 are important when oxygen and glucose levels are limiting, as often occurs in solid tumours [110].

## FBPase antagonizes cancer chemoresistance

Gemcitabine is the first-line chemotherapy for pancreatic cancer [111]. However, gemcitabine fails to significantly improve prognosis of pancreatic carcinoma patients due to acquisition of chemoresistance in patients. It is well documented that gemcitabine treatment results in undesirable activation of the RAS-RAF-MAPK pathway [112]. IQ motif-containing GTPase-activating protein 1 (IQGAP1) is a MAPK scaffold that directly regulates the activation of RAF, MEK, and extracellular signalling-regulated kinases (ERKs) [113]. In pancreatic cancer, FBP1 expression impeded gemcitabine-induced ERK activation through inhibition of the IQGAP1-ERK1/2 signalling axis in a manner independent of its enzymatic activity. Co-treatment of FBP1-derived small peptide inhibitor FBP1 E4 enhanced the anti-cancer efficacy of gemcitabine in pancreatic cancer [27]. However, the molecular mechanisms by which FBP1 antagonizes cancer chemoresistance in pancreatic cancer warrant further investigation. Moreover, whether FBP1 exerts similar effect in chemoresistance in other cancer types needs to be elucidated.

## Regulation of aberrant expression of FBP1 in cancer

The mechanisms for the aberrant expression of FBP1 are mainly focused on the epigenetic regulation of the FBP1 promoter. However, recently, ubiquitin-mediated degradation and copy number loss of FBP1 were also shown to explain the loss of FBPase in cancer (Table 2).

**Table 2:** Regulation of aberrant expression of FBP1 in cancer

Regulator	Interaction	Reference(s)
LSD1	Promoter methylation	[114]
NF-kappaB	Promoter methylation	[26]
NPM1	Promoter methylation	[28]
Snail-G9a-Dnmt1	Promoter methylation	[22]
ZEB1	Promoter methylation	[13]
HDAC1/2	Histone deacetylation	[81]
Copy number loss	Genomic alterations	[15, 86]
TRIM28	Ubiquitination degradation	[80]

### Epigenetic changes

Epigenetic changes, including DNA methylation and histone modifications, have been shown to alter patterns of gene expression and to be involved in carcinogenesis [115]. The methylation of the FBPase promoter has been observed in various cancers [13, 15, 17, 22, 25, 26, 75, 114]. NF-ΚB functioning downstream of the Ras pathway promoted the epigenetic downregulation of FBP1 in gastric cancer [26]. The Snail-G9a-Dnmt1 complex, which is critical for E-cadherin promoter silencing and the corresponding increase in H3K9me2 and DNA methylation, was also required for the promoter-methylation of FBP1 in BLBC [22]. Lysine (K)-specificdemethylase 1A (LSD1)-mediated demethylation of H3K4me2 at FBP1 promoters suppressed FBP1 expression in HepG2 cells [114]. Zinc finger E-box-binding homeobox 1 (ZEB1) bound to the FBP1 promoter to enhance DNA methylation in lung cancer [13]. NPM1 bound directly to the FBP1 promoter region to suppress the expression of FBP1 in pancreatic cancer [28]. Demethylation with 5-aza-deoxycytidine (5AZA), Ras inhibitor or LSD inhibitor restored FBP1 expression, implying that hypermethylation is directly responsible for the loss or downregulation of FBP1 expression [13, 26, 75]. A study has shown that additional inhibition of histone deacetylase inhibitor further increased FBPase expression compared to 5aza alone [75], suggesting that histone deacetylation may contribute synergistically to the silencing of FBPase. In line with this finding, histone deacetylase 1 (HDAC1) and HDAC2 together induced the suppression of FBP1 expression by decreasing histone H3 lysine 27 acetylation (H3K27Ac) of the FBP1 enhancer in HCC. Treatment with HDAC inhibitors or knockdown of HDAC1 and/or HDAC2 restored FBP1 expression [81].

## Copy number loss

Copy number loss of FBP1 was observed in ccRCC cases [86]. Copy number loss of FBP1 was significantly associated with lower FBP1 expression in HCC [15]. These data indicated that genomic alterations were also responsible for FBP1 inhibition.

## Ubiquitin-mediated degradation

Recently, the E3 ubiquitin ligase TRIM28 has been found to play a critical role in regulating FBP1 protein levels through a post-translational mechanism in HCC. TRIM28 was found to directly bind to and promote the ubiquitination and degradation of FBP1. MAGE-A3 and MAGE-C2, which are known to specifically bind to TRIM28 [94, 116], can enhance TRIM28-dependent degradation of FBP1 by forming ubiquitin ligase complexes with TRIM28 [80].

## Chemical inhibitors utilized to restore FBP1 expression

**Fig. 4:**
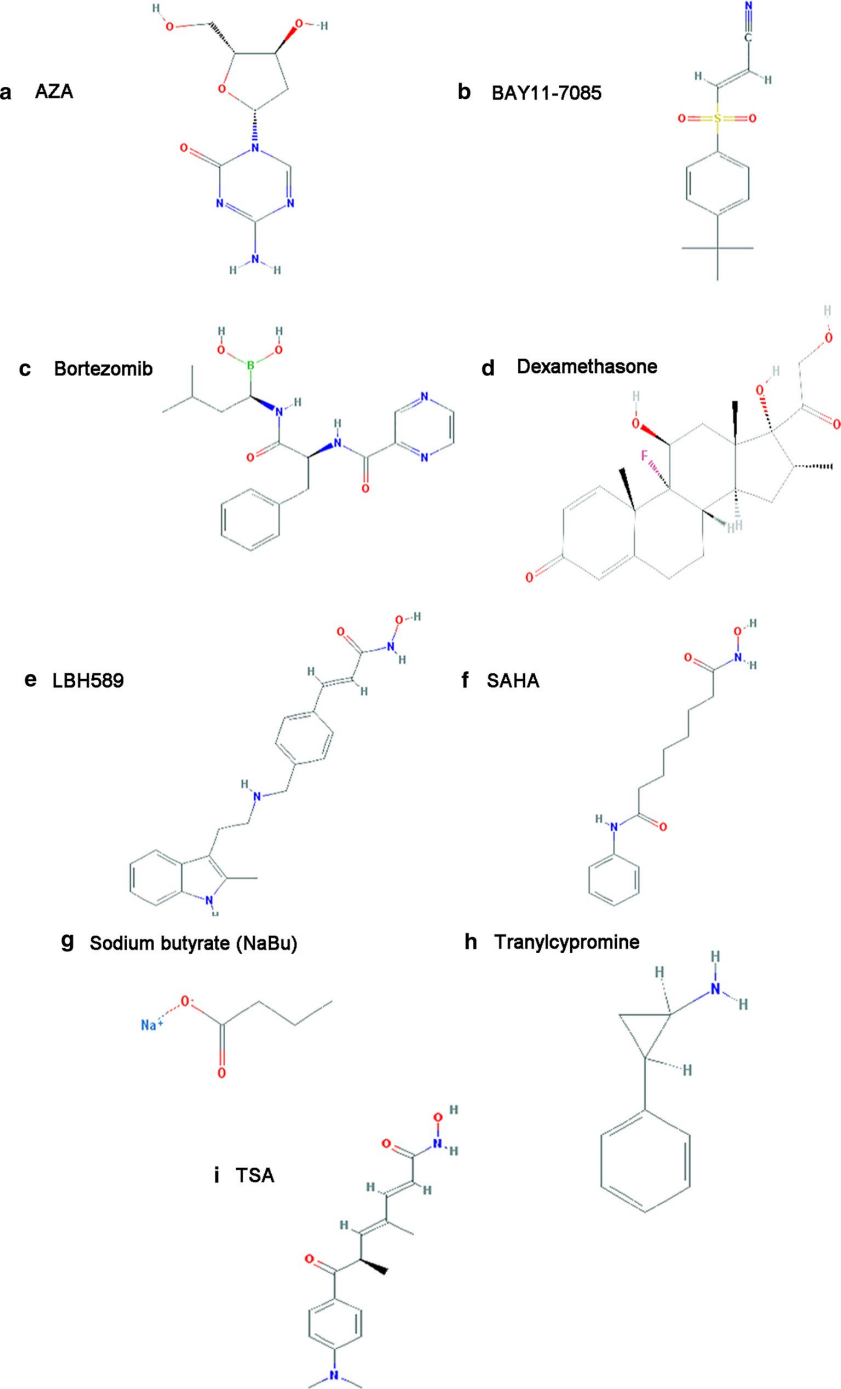
Structures of chemical inhibitors restoring FBPase. **a** 5-Aza-deoxycytidin (5AZA) [117], **b** BAY11-7085 [118], **c** bortezomib [119], **d** dexamethasone [120], **e** LBH589 [121], **f** SAHA [122], **g** sodium butyrate (NaBu) [123], **h** tranylcypromine (TCP) [124], **i** trichostatin A (TSA) [125]

Several chemical inhibitors have been shown to successfully restore the expression of FBP1. Drugs targeting promoter methylation, HDAC and the upstream regulator of FBPase, have been proven efficacious. Their interactions are detailed below (Fig. 4).

Recently, 5AZA, a hypomethylating agent [126], together with HDAC inhibitors, such as trichostatin A (TSA), sodium butyrate (NaBu) [127], SAHA [128] and LBH589 [129], have been approved by the Food and Drug Administration (FDA) of the USA for cancer therapy in myelodysplastic syndrome (MDS) and acute myeloid leukaemia (AML) [130]. A combination of DNA methyltransferase inhibitors and HDAC inhibitors has also shown promising synergistic effects in the treatment of MDS, AML and non-Hodgkin’s lymphoma [131]. Treatment of cells with 5AZA resulted in a significant increase in the expression of FBPase mRNA in breast cancer, gastric cancer and lung cancer [13, 25, 75], while treatment with sodium butyrate, SAHA and LBH589 upregulated FBP1 protein and mRNA expression in HCC [81]. Restoration of FBP1 expression by HDAC inhibitors led to a switch from glycolysis to gluconeogenesis, altered energy metabolism and inhibition of tumour growth [81]. All these data indicated that the silencing of FBP1 can be a target of methyltransferase inhibitors and HDAC inhibitors for the potential treatment of cancer. However, we are still a long way from the clinical application of these epigenetic drugs for solid tumours [132].

LSD1, the first histone demethylase identified, converts active H3K4me2/3 to the less active H3K4me1 mark, leading to gene activation [133]. Meanwhile, LSD1 also converts the inactive H3K9me3 into the less repressive H3K9me1 or H3K9me2 marks, thereby leading to gene derepression [134]. Thus, the functional outcome of LSD1 activity depends on the balance between the modification of H3K4me2/3 or H3K9me3. Tranylcypromine (TCP) is a potent inhibitor of the demethylation activity of LSD1 [135]. FBP1 was robustly and quickly induced by TCP treatment in HepG2 cells [114]. However, before using LSD1 inhibitors, their pleiotropic actions should be taken into careful consideration.

Bortezomib, a 26S proteasome inhibitor, has been approved by the FDA for the treatment of multiple myeloma and mantle cell lymphoma [136–139]. In HCC, a TRIM28-induced decrease in FBP1 protein levels (but not mRNA levels) was completely inhibited by bortezomib treatment. Meanwhile, bortezomib-induced decreases in glucose consumption, lactate levels and cell growth inhibition were largely diminished by the knockdown of FBP1. These data indicated that bortezomib could regulate the Warburg effect by inhibiting the proteasome-dependent degradation of FBP1 [80], which might be harnessed in combination therapy [140], but not bortezomib monotherapy, for cancer [141].

In addition, drugs targeting upstream regulators of FBPase have shown efficacy in restoring FBPase expression. Ras, the first oncogene isolated from human tumour cells, was aberrantly activated in many cancers [142]. In gastric cancer, Ras-induced FBP1 downregulation was reversed after the inhibition of NF-ΚB activity by either a chemical inhibitor of NF-ΚB, BAY11-7085, or a genetic suppressor of NF-ΚB, IkB-alpha M [26, 143]. Dexamethasone, an active form of synthesized glucocorticoids, restored the expression of gluconeogenesis genes, including FBP1, thereby antagonizing the Warburg effect and leading to therapeutic efficacy in the treatment of hepatocarcinoma [89]. Nevertheless, considering the complex regulatory networks of these regulators, adverse effects and toxicity caused by poor specificity may always be potential problems in their clinical applications.

## Conclusions and perspectives

Research on metabolic reprogramming in cancer is progressing at an unprecedented pace. FBPase, one of the rate-limiting enzymes responsible for gluconeogenesis, is usually found to be downregulated in many cancers and is treated as a tumour-suppressor gene. Meanwhile, these results should be interpreted with caution due to only one to two publications in some cancer types such as in colon cancer, pancreatic cancer and small intestinal neuroendocrine tumor. In most cancers, FBPase goes far beyond its enzymatic function, as it is located inside the cell nucleus and is efficacious through a catalytic-activity-independent mechanism via direct interaction with other genes. In our opinion, to maintain rapid proliferation and differentiation, all cancer cells will confront a microenvironmental energy crisis, including the intrinsic shortage of metabolic substrates and energy as well as the external deterioration of the microenvironment due to factors such as hypoxia, acidosis and hypoglycaemia. To overcome all these unfavourable growth conditions, cancer cells have to reprogram their metabolic and epigenetic phenotypes through the activation of oncogenes and inactivation of tumour-suppressor genes, altering the pattern of epigenetic modification and leading to the aberrant expression of numerous genes, including those involved in metabolic rate-limiting, metastasis or differentiation. Therefore, it is not surprising to find an aberrant epigenetic modification of FBPase in cancer. Furthermore, it is also not surprising to find a close correlation between FBPase expression and transcriptional factors such as HIF, EMT transcriptional factors and cancer differentiation. However, the complete mechanism underlying the interplay of FBPase and other genes is complicated and unclear. Does noncoding RNA play an important role in the regulation of FBPase expression? What is the meaning and intrinsic mechanism of the interplay between aberrant FBPase and other cancer hallmarks? Investigating the role of FBPase in CSCs and apoptosis, for example, will promote our understanding of cell differentiation and programmed cell death and reveal new clues for combating cancer.

Several problems remain to be solved for the use of FBPase as a target in cancer treatment. One main obstacle is the identification of distinct regulatory markers exclusive to aberrant FBPase. Without this knowledge, drugs targeting aberrant FBPase with higher specificity but fewer side effects can’t be found or designed. Another problem is identifying the optimal timing for the use of FBPase-targeting drugs, as these drugs may have completely different effects at different stages of progression of different types of cancer. These drugs may induce a switch from aerobic glycolysis to OXPHOS and inhibit the aggressive phenotypes of some cancers; meanwhile, in other circumstances, they may help promote the survival of cancer cells and sustain metastasis. Co-treatment of drugs targeting FBPase with conventional chemotherapy is promising, as expression of FBPase promotes differentiation and apoptosis and inhibits the chemoresistance of cancer cells. However, the efficacy of cancer drugs targeting FBPase warrants further investigation in the real world.

## Abbreviations

5AZA: 5-aza-deoxycytidin
AML: acute myeloid leukaemia
AMP: adenosine monophosphate
AKT: protein kinase B
ATP: adenosine triphosphate
BCAAs: branched chain amino acids
BLBC: basal-like breast cancer
BNIP3L/NIX: adenovirus E1B 19 kDa interacting protein 3-like
cAMP: cyclic adenosine monophosphate
ccRCC: clear cell renal cell carcinoma
CBP: CREB-binding protein
CREB: cAMP response element-binding protein
CRTC2: CREB coactivator
CSCs: cancer stem cells
EpCAM: epithelial cell adhesion molecule
H3K27Ac: histone H3 lysine 27 acetylation
HDAC: histone deacetylase
Dnmt1: DNA methyltransferase 1
ECAR: extracellular acidification rate
ERKs: extracellular signalling-regulated kinases
F1,6BP: fructose-1,6-bisphosphate
F2,6BP: fructose-2,6-bisphosphate
FOXO: forkhead box O protein
FOX O3: forkhead box O3 protein
F6P: fructose-6-phosphate
FBPase: fructose-1,6-bisphosphatase
FDG-PET: 18F-deoxyglucose positron emission tomography
G3P: glycerol-3-phosphate
GCs: gastric carcinomas
GLUT: glucose transporter
GCGR: glucagon receptor
GR: glucocorticoid receptor
GREs: glucocorticoid responsive elements
GSIS: glucose-induced insulin secretion
HCC: hepatocellular carcinoma
HGP: hepatic glucose production
HIF1: hypoxia-inducible factor-1
HK: hexokinase
IQGAP1: IQ motif-containing GTPase-activating protein 1
LDHA: lactate dehydrogenase A
LSD1: lysine (K)-specificdemethylase 1A
MAPK: mitogen-activated protein kinase
MDS: myelodysplastic syndrome
MMP7: matrix metalloproteinase-7
mTOR: mammalian target of rapamycin
NAD+: nicotinamide adenine dinucleotide
NADP: nicotinamide adenine dinucleotide phosphate
NADPH: triphosphopyridine nucleotide
OCR: oxygen consumption rate
OXPHOS: oxidative phosphorylation
PARP: poly ADP-ribose polymerase
PEP: pyruvatel/phosphoenolpyruvate
PFK: phosphofructokinase
PFK1: phosphofructokinase-1
PFK-2/FBPase-2: 6-phosphofructo-2-kinase/fructose-2,6-bisphosphatase
PI3K: phosphoinositide 3-kinase
PI: propidium iodide
PKA: protein kinase A
PK: pyruvate kinase
PKM2: pyruvate kinase M2
ROS: reactive oxygen species
SOD: superoxide dismutase
SIRM: stable isotope-resolved metabolomic
T2DM: type 2 diabetes
TCA: tricarboxylic acid
TCF4: transcription factor 4
TCP: tranylcypromine
TIGAR: TP53-induced glycolysis and apoptosis regulator
TNBC: triple-negative breast cancer
TSA: trichostatin A
UDPG: uridine diphosphate glucose
ZEB1: zinc finger E-box-binding homeobox 1
